# Variations in ribosomal DNA copy numbers in a genome of *Trichophyton interdigitale*

**DOI:** 10.1111/myc.13163

**Published:** 2020-09-02

**Authors:** Tomoyuki Iwanaga, Kazushi Anzawa, Takashi Mochizuki

**Affiliations:** ^1^ Department of Dermatology Kanazawa Medical University Ishikawa Japan

**Keywords:** dermatophytes, quantitative real‐time PCR, ribosomal DNA, *Trichophyton interdigitale*

## Abstract

**Background:**

Ribosomal DNA (rDNA) reportedly has multiple copies in the fungal genome. The internal transcribed spacer (ITS) region in rDNA is useful for investigating relationships between close taxonomic relatives. Thus, ITS has been widely used as a target gene in medical mycology for the detection of pathogenic fungi and identification of fungal species. However, the rDNA copy number in a genome of *Trichophyton interdigitale*, the pathogen causing dermatophytosis, currently remains unknown.

**Objective:**

Clarification of the rDNA copy number in a genome of *T. interdigitale*.

**Methods:**

rDNA copy numbers in 64 clinical isolates of *T. interdigitale* were examined using quantitative real‐time PCR (qPCR) with the absolute quantitative method targeting TruMDR2, a single‐copy control gene and the ITS region in rDNA.

**Results:**

The copy numbers of the rDNA subunit varied among the 64 strains tested, from 24 to 116 copies per genome. The average rDNA copy number ± standard deviation was 59 ± 16. No correlations were observed between the rDNA copy number and colony colour, colony morphology or molecular type of the non‐transcribed spacer region in rDNA. Experiments on rDNA copy numbers obtained from independent colonies of each strain in single‐spore cultures revealed that the copy number was homogeneous within each strain.

**Conclusion:**

This is the first study to estimate copy numbers of the rDNA subunit in a genome of *T. interdigitale*. The rDNA copy number of *T. interdigitale* varied among the strains tested and was homogeneous within each strain.

## 1 INTRODUCTION

*Trichophyton interdigitale* ^1^ is a major pathogen that causes dermatophytosis, such as tinea pedis and tinea unguium.^2^, ^3^ The gold standard for the diagnosis or evaluation of the therapeutic outcome of dermatophytosis is a direct KOH examination and fungal culture. However, difficulties are associated with its identification due to morphological similarities, variabilities and polymorphisms in dermatophytes. Therefore, in addition to these conventional diagnostic methods, molecular biological techniques using PCR for the diagnosis of dermatophytosis have been developed to detect and identify the fungal strain from clinical isolates and specimens affected by dermatophytes.^4^, ^5^ Among them, internal transcribed spacer (ITS) region, located between the 18S and the 28S ribosomal DNA (rDNA), targeted PCR method, such as PCR‐restriction fragment length polymorphism (RFLP),^6^, ^7^, ^8^ have been widely applied to the identification of dermatophytes species. Because 18S and 28S rDNA are useful PCR targets because of their sequence conservation in dermatophytes, which has allowed for the use of universal primers that enable the amplification of targets from unknown dermatophyte species.^9^ A second advantage of targeting rDNA is that the DNA sequence of the ITS region considerably varies between fungal species and exhibits adequate variability for differentiating between closely related dermatophyte species.^10^, ^11^, ^12^, ^13^, ^14^ Furthermore, a genome is known to have multiple copies of rDNA. Thus, the detection of rDNA is more advantageous than other single‐copy genes.

rDNA repeats in the fungal genome were previously reported to be between 28 and 118 using six fungal species,^15^ and between 14 and 1442 using 91 species.^16^ These data contribute to estimations of the amount of pathogenic fungi in affected specimens. However, the copy number in a genome (copy number) of *T. interdigitale* and whether all strains have the same number of rDNA subunits currently remain unclear. According to previous studies on other fungi, the rDNA copy number varied among strains and ranged between 56 and 225 copies per genome in four strains of *Leptosphaeria maculans*,^17^ between 54 and 511 copies per genome in 36 strains of *Saccharomyces cerevisiae*,^18^ and between 38 and 91 copies per genome in eight strains of *Aspergillus fumigatus*.^19^ Furthermore, the rDNA copy number of *A. fumigatus* remained stable under various environmental conditions.^19^

In the present study, we investigated rDNA copy numbers in 64 isolates of *T. interdigitale* by qPCR. The homogeneity of rDNA copy numbers in each strain of *T. interdigitale* was assessed by sampling genomic DNA from independent colonies in single‐spore cultures.

## 2 MATERIALS AND METHODS

### 2.1 Ethics statement

The authors confirm that the ethical policies of the journal, as noted on the journal's author guidelines page, have been adhered to. No ethical approval was required as the research in this article related to micro‐organisms.

### 2.2 Fungal strains

All 64 clinical isolates of *T. interdigitale* were obtained from the culture collection of Kanazawa Medical University (KMU). They were identified by colony morphology, a PCR‐RFLP analysis^7^ and the sequence analysis of the ITS region in rDNA. The ITS sequence was determined using the primer of ITS4 and ITS5,^9^ BigDye™ Terminator v3.1 Cycle Sequencing Kit (Applied Biosystems) and 3500xL Genetic Analyzer (Applied Biosystems). The molecular type of the non‐transcribed spacer (NTS) regions of rDNA accumulates high degrees of sequence variations and is available for the detection of these strain variations. In our previous study, the NTS types of the 64 clinical strains used in the present study were classified into 15 molecular types.^20^

### 2.3 Primer design

The primers, ITSF and ITSR (Table 1), were designed based on the nucleotide sequence of the ITS region in rDNA, as previously reported.^21^ The primer pair specifically detects *Trichophyton* species and *Microsporum* species. TruMDR2, which encodes an ATP‐binding cassette (ABC) transporter related to multidrug resistance, was selected as a single‐copy control because it was reported to be a single‐copy gene in a genome of *Trichophyton rubrum* ^22^ and was a single copy confirmed by the whole genome sequencing (WGS) of two strains of *T. interdigitale* (AOKS00000000.1 and AOKY00000000.1, GeneBank^®^). The primers, MDRF and MDRR (Table 1), were designed based on the published sequence data of TruMDR2 in *T. rubrum* (AF291822, GenBank^®^), and WGS data obtained from *T. interdigitale* (AOKS00000000.1 and AOKY00000000.1, GenBank^®^).

**Table 1:** Primers used in the present study

Primer sequences
ITSF 5′‐AGCCCGGCTTGTGTGATG‐3′
ITSR 5′‐CATTCGCCTAGGAAGCCG‐3′
MDRF 5′‐ ATCAAGGAACGGATCGTCAG‐3′
MDRR 5′‐ AATAGCAATACGCTGCTTCTG‐3′

### 2.4 Standard curve of quantitative real‐time PCR (qPCR)

Template DNA for the quantitative analysis was prepared from plasmid DNA, constructed using a TOPO^®^ TA Cloning^®^ Kit (Invitrogen). The ITS and MDR region of *T. interdigitale* was amplified using the primer pairs of ITS and MDR (Table 1), and cloned into the plasmid vector pCR2.1^®^‐TOPO^®^. The copy number of templates for standard curves was calculated from the size of the cloned plasmid and DNA concentration. Standard curves were generated using 10‐fold serial dilutions, yielding samples containing 10^2^ to 10^7^ copies of ITS DNA per reaction. Assays to generate standard curves were performed using the 7900HT Fast Real‐Time PCR System (Applied Biosystems) and SYBR^®^ Green PCR Kit (Qiagen) in a total volume of 25 μL containing 5 μL of template DNA, 12.5 μL of SYBR^®^ Green Master Mix and 1 μL of each primer (ITSFR and MDRFR, 12.5 μmol/L). The amplification protocol consisted of 15 minutes of denaturation at 95°C, followed by 40 cycles of denaturation at 95°C for 10 seconds, annealing at 60°C for 30 seconds and extension at 72°C for 10 seconds, with the addition of a dissociation stage for a subsequent melting curve analysis. Amplification efficiency and the correlation coefficient (*R* ^2^) between the copy number of the template and cycle threshold (Ct) were assessed by linear approximation.

### 2.5 DNA extraction and quantification by qPCR

DNA was extracted from all 64 clinical isolates by a mini‐prep method^12^, ^23^ with slight modifications. Briefly, a small amount of the fungal mat was immersed in 200 μL of lysis buffer (200 mmol/L Tris‐HCl (PH 7.5), 0.5% (w/v) sodium dodecyl sulphate, 25 mmol/L ethylenediaminetetraacetate and 250 mmol/L NaCl) and ground with a conical grinder. After heating at 100°C for 5 minutes, 100 μL of 3.0 mol/L sodium acetate was added to each homogenate, which were then incubated at 20°C for 20 minutes, followed by centrifugation at 12 000 *g* for 5 minutes. DNA samples were precipitated from each supernatant with an equal volume of isopropanol. Each precipitate was washed with 70% ethanol, dried and dissolved in 50 μL of distilled water.

Aliquots of 5 μL from DNA were used as the template for qPCR. qPCR was performed under the same conditions as described above.

### 2.6 Calculation of rDNA copy numbers

The rDNA copy numbers of each strain were calculated as the ratio of the template quantity for rDNA to that for TruMDR2. The rDNA copy number was obtained using the following equation: rDNA copy number = (total copies of ITS)/(total copies of TruMDR2).

### 2.7 Homogeneity of rDNA copy numbers

A single‐spore culture was performed by the dilution method using five strains of *T. interdigitale* (KMU 6498, 6510, 6524, 6540 and 6547). A small amount of the fungal mat was suspended in physiological saline, and a conidial suspension was obtained by filtration using a cell strainer (Becton Dickinson and Company, Tokyo, Japan). Each suspension was gradually diluted, and an aliquot of each dilution was inoculated onto the Sabouraud dextrose agar plate. More than three colonies of the single‐spore culture were selected from each strain, and the rDNA copy number of each colony was assessed by qPCR described above.

### 2.8 Statistical analysis

Student's t test was used to compare the rDNA copy number of each strain with colony colour and colony morphology. A one‐way ANOVA followed by the Tukey‐Kramer post hoc analysis was performed to compare rDNA copy numbers and the NTS molecular type in the case of more than three strains of each NTS type.

## 3 RESULTS

### 3.1 Standard curve of qPCR

**Figure 1:**
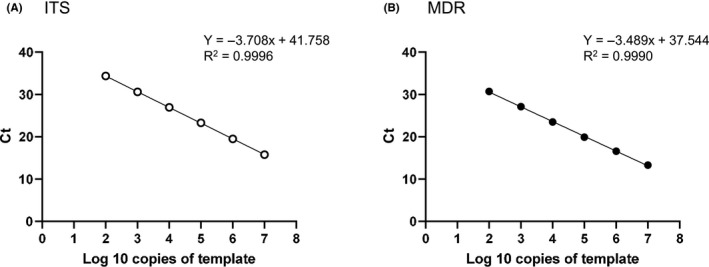
Standard curve for cycle threshold (Ct) versus template DNA copies by qPCR. Serial dilutions of plasmid DNA containing the cloned internal transcribed spacer (ITS) region of rDNA (A) and TruMDR2 (MDR) (B) in *Trichophyton interdigitale* were tested by qPCR using sets of ITS and MDR primers. Each point represents the mean of triplicate measurements. The standard deviations of each point were too small to show in the graph

A standard curve (ITS primer: *Y* = −3.708*x* + 41.758, *R* ^2^ = 0.9996, MDR primer: *Y* = −3.489*x* + 37.544, *R* ^2^ = 0.9990) was obtained in a linear range of 10^2^ to 10^7^ copies for the Ct value versus the copy number of cloned ITS and MDR of *T. interdigitale* (Figure 1). As a result of the sequencing of PCR amplicons using ITS and MDR primers, the sizes of amplicons targeting parts of the ITS and TruMDR2 regions were 107 and 138 bp, respectively, in *T. interdigitale*. These sequences completely matched the registered sequences (ITS: FM986778, MDR: AOKS00000000.1 and AOKY00000000.1, GeneBank^®^).

### 3.2 Assessment of rDNA copy numbers

**Figure 2:**
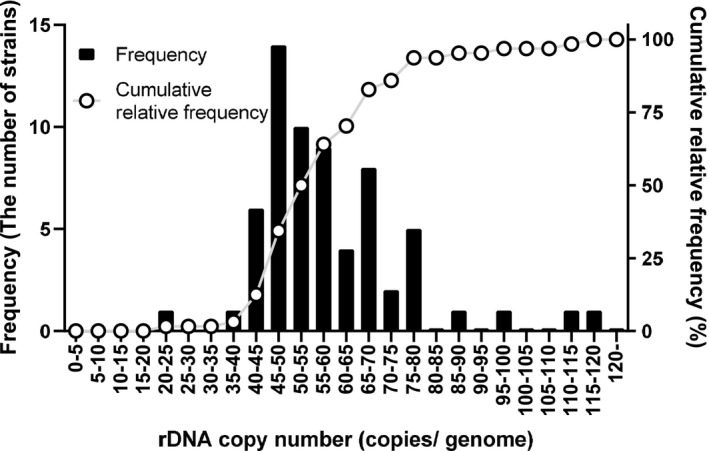
A histogram of rDNA copy numbers in a genome of *Trichophyton interdigitale*. rDNA copy numbers in the genomes of 64 strains were calculated by the ITS regions of rDNA and TruMDR2 (MDR) targeted by qPCR. MDR was used as the target of a single‐copy gene. The frequency of every 5 rDNA copy numbers and the cumulative relative frequency were shown in the graph. The data obtained showed a range of 24 to 116 copies, with an average and standard deviation (S. D.) of 59 copies ± 16 per genome. These results showed that rDNA copy numbers were strain‐specific and markedly varied for every strain

The rDNA copy numbers of the 64 *T. interdigitale* isolates were evaluated using TruMDR2 as the reference single‐copy gene. The data obtained revealed a range of 24 to 116 copies, with an average and standard deviation (S. D.) of 59 copies ± 16 per genome, and were shown on a histogram (Figure 2). These results demonstrated that rDNA copy numbers were strain‐specific and markedly varied for every strain.

### 3.3 Relationships between rDNA copy numbers and strain features

**Figure 3:**
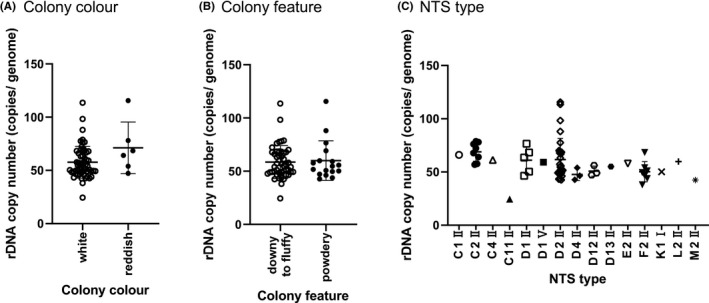
The relationship between rDNA copy numbers and characteristics of *Trichophyton interdigitale* strains. Sixty‐four strains of *T. interdigitale* were divided by colony colour, colony morphology and the molecular type of the non‐transcribed spacer (NTS) region in rDNA. A, Fifty‐eight strains showed white colonies, while 6 were reddish. B, Forty‐seven strains showed a downy to fluffy morphology and 17 were powdery. C, Sixty‐four strains of *T. interdigitale* were classified into 15 NTS molecular types. The NTS types of C1Ⅱ, C2Ⅱ, C4Ⅱ, C11Ⅱ, D1Ⅱ, D1Ⅴ, D2Ⅱ, D4Ⅱ, D12Ⅱ, D13Ⅱ, E2Ⅱ, F2Ⅱ, K1Ⅰ, L2Ⅱ and M2Ⅱ comprised 1, 8, 1, 1, 5, 1, 29, 3, 3, 1, 1, 7, 1, 1 and 1 strains, respectively. The relationship between rDNA and colony colour or colony morphology was analysed using the Student's t test, and a one‐way ANOVA followed by the Tukey‐Kramer post hoc analysis was used to examine the NTS type. rDNA copy numbers did not correlate with colony colour, colony morphology or the NTS type

The rDNA copy number, colony colour, colony morphology and NTS type of the 64 clinical isolates are shown in Table 2. Regarding the results of observations of colony features, the colony colour of 58 strains was white, while that of 6 strains was reddish, and the colony morphology of 47 strains was downy to fluffy, while that of 17 strains was powdery. The 64 strains of *T. interdigitale* were classified into 15 NTS molecular types. The NTS types of C1Ⅱ, C2Ⅱ, C4Ⅱ, C11Ⅱ, D1Ⅱ, D1Ⅴ, D2Ⅱ, D4Ⅱ, D12Ⅱ, D13Ⅱ, E2Ⅱ, F2Ⅱ, K1Ⅰ, L2Ⅱ and M2Ⅱ comprised 1, 8, 1, 1, 5, 1, 29, 3, 3, 1, 1, 7, 1, 1 and 1 strains, respectively. The relationships between rDNA and colony colour, colony morphology and NTS type were analysed statistically. The rDNA copy number did not correlate with colony colour, colony morphology or the NTS type (Figure 3A‐C).

**Table 2:** Summary of *Trichophyton interdigitale* isolates

KMU No.	Origin	Clinical features	Colony features		NTS type	rDNA copy number
			Colour^a^	Morphology^a^		
6492	T.P.^b^	Scale, vesicles	White	Powdery	D2Ⅱ	51
6493	T.P.	—	White	Downy to fluffy	D2Ⅱ	49
6494	T.P.	Hyperkeratosis	White	Powdery	D2Ⅱ	88
6495	T.P.	Scale, vesicles	White	Downy to fluffy	D2Ⅱ	57
6496	T.P.	Scale	White	Downy to fluffy	D1Ⅱ	64
6497	T.P.	Scale	White	Downy to fluffy	C2Ⅱ	72
6498	T.P.	Scale, hyperkeratosis	White	Downy to fluffy	F2Ⅱ	50
6499	T.U.^c^	Superficial cloudy	White	Downy to fluffy	D1Ⅱ	68
6500	T.P.	Scale	White	Downy to fluffy	C2Ⅱ	57
6501	T.U.	Nail cloudy	White	Downy to fluffy	C2Ⅱ	68
6502	T.P.	Vesicle, interdigital maceration	White	Downy to fluffy	D2Ⅱ	69
6503	T.P.	Scale	White	Downy to fluffy	F2Ⅱ	54
6504	T.P.	Hyperkeratosis	White	Downy to fluffy	C2Ⅱ	76
6505	T.P.	Hyperkeratosis	White	Downy to fluffy	D12Ⅱ	49
6506	T.P.	—	White	Powdery	D2Ⅱ	55
6507	T.P.	—	White	Powdery	D1Ⅴ	59
6508	T.P.	Bullae	White	Powdery	D4Ⅱ	43
6509	T.P.	Interdigital maceration, erosion	White	Downy to fluffy	D2Ⅱ	68
6510	T.P.	Scale, pustules	White	Downy to fluffy	D1Ⅱ	50
6511	T.P.	Scale, vesicles	Red	Powdery	D2Ⅱ	116
6512	T.P.	Scale	White	Downy to fluffy	D2Ⅱ	49
6513	T.P.	Scale	White	Powdery	D2Ⅱ	69
6514	T.P.	Scale	Red	Downy to fluffy	F2Ⅱ	68
6515	T.P.	Scale, vesicles	Red	Downy to fluffy	C2Ⅱ	64
6516	T.P.	Scale, nail cloudy	White	Downy to fluffy	C2Ⅱ	79
6517	T.P.	‐	Red	Downy to fluffy	C2Ⅱ	78
6518	T.P.	Pustules	White	Downy to fluffy	D2Ⅱ	43
6519	T.P.	Scale, papules	White	Downy to fluffy	D2Ⅱ	49
6520	T.P.	Scale, vesicles	White	Downy to fluffy	C11Ⅱ	24
6521	T.P.	Scale, vesicles	White	Powdery	D2Ⅱ	44
6522	T.P.	Scale, pustules	White	Downy to fluffy	F2Ⅱ	44
6523	T.P.	Scale, vesicles	White	Downy to fluffy	D2Ⅱ	43
6524	T.U.	KOH‐, nail cloudy	White	Downy to fluffy	D12Ⅱ	56
6525	T.P.	Scale, vesicle, hyperkeratosis	White	Downy to fluffy	F2Ⅱ	51
6526	T.P.	Scale	White	Downy to fluffy	D13Ⅱ	55
6527	T.P.	Scale	Red	Downy to fluffy	D4Ⅱ	54
6528	T.P.	Scale	White	Downy to fluffy	F2Ⅱ	47
6529	T.P.	Pustules	White	Powdery	D2Ⅱ	50
6530	T.P.	Bullae	White	Powdery	D2Ⅱ	52
6531	T.P.	Scale, pustules	White	Downy to fluffy	E2Ⅱ	58
6532	T.P.	Scale	White	Downy to fluffy	F2Ⅱ	38
6533	T.P.	Scale, pustules	White	Downy to fluffy	D2Ⅱ	48
6534	T.P.	Bullae	White	Downy to fluffy	C1Ⅱ	66
6535	T.U.	Nail cloudy	Red	Powdery	D12Ⅱ	47
6536	T.P.	—	White	Downy to fluffy	D2Ⅱ	51
6537	T.P.	Scale, pustules	White	Powdery	D1Ⅱ	77
6538	T.P.	—	White	Downy to fluffy	D2Ⅱ	114
6539	T.P.	Bulla, trichophytid +	White	Downy to fluffy	D2Ⅱ	48
6540	T.P.	Scale	White	Powdery	L2Ⅱ	60
6541	T.P.	Scale	White	Powdery	D2Ⅱ	56
6542	T.P.	Scale, pustules	White	Downy to fluffy	M2Ⅱ	42
6543	T.P.	Scale	White	Downy to fluffy	D1Ⅱ	46
6544	T. manus	Bullae, erosion	White	Downy to fluffy	D2Ⅱ	52
6545	T.P.	KOH ‐, combine keratosis	White	Downy to fluffy	D2Ⅱ	78
6546	T.P.	Scale, vesicles	White	Powdery	C2Ⅱ	58
6547	T.U.	KOH ‐, nail cloudy, nail hypertrophy	White	Downy to fluffy	D2Ⅱ	70
6549	T.P.	Thick hyperkeratosis, scale	White	Powdery	K1Ⅰ	50
6550	T.P.	—	White	Downy to fluffy	D2Ⅱ	98
6551	T.P.	Hyperkeratosis, interdigital maceration	White	Downy to fluffy	D4Ⅱ	46
6552	T.P.	Scale, interdigital maceration	White	Downy to fluffy	D2Ⅱ	63
6553	T. cruris	Erythema gyratum, tinea pedis +, tinea unguium +	White	Powdery	D2Ⅱ	46
6554	T.U.	Nail cloudy	White	Downy to fluffy	C4Ⅱ	61
6555	T.P.	Hyperkeratosis, scale, nail cloudy, Woods light +	White	Downy to fluffy	D2Ⅱ	67
6556	T.P.	Scale, vesicles	White	Downy to fluffy	D2Ⅱ	49
					Average	59

^a^ Subculture on potato dextrose agar slants.

^b^ Tinea pedis.

^c^ Tinea unguium.

### 3.4 Homogeneity of rDNA copy numbers

**Figure 4:**
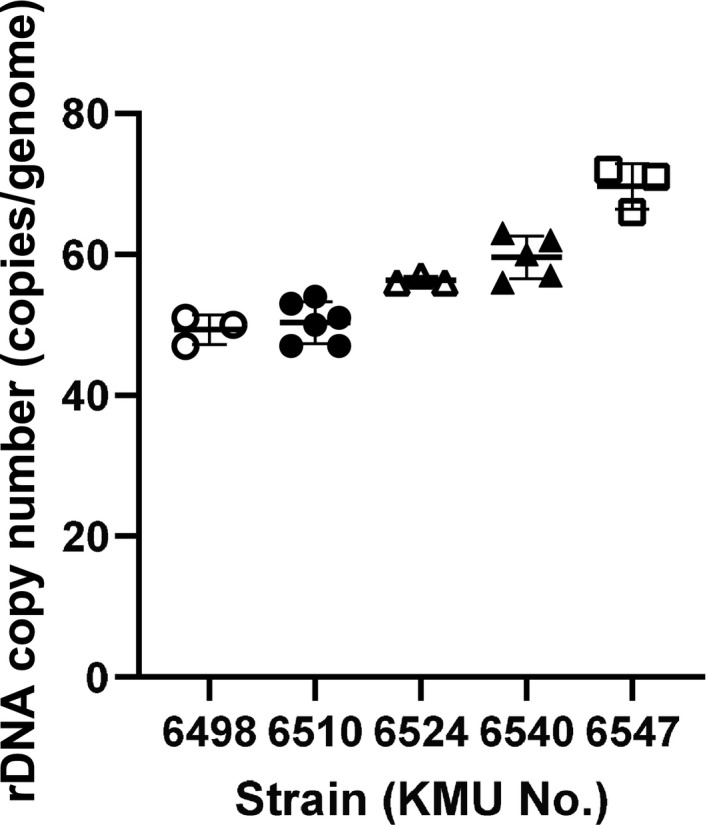
Homogeneity of the rDNA copy number in a genome of *Trichophyton interdigitale*. Homogeneity evaluations of rDNA copy numbers using a single‐spore culture of *T. interdigitale* strains. rDNA copy numbers were 50 ± 2.0, 50 ± 2.7, 56 ± 0.6, 60 ± 2.7 and 70 ± 2.5 (mean ± S. D.) in the genomes of KMU 6498, 6510, 6524, 6540 and 6547, respectively. The coefficients of variation (CV, CV = S. D./mean) of the rDNA copy number in a single strain were calculated to be 4.0, 5.3, 1.0, 4.5 and 3.6, respectively. rDNA copy numbers were assessed from more than three colonies of each strain

rDNA copy numbers from the different single‐spore cultures were calculated as 50 ± 2, 50 ± 2.7, 56 ± 0.6, 60 ± 2.7 and 70 ± 2.5 (mean ± S. D.) in the genomes of KMU 6498, 6510, 6524, 6540 and 6547, respectively (Figure 4). The coefficient of variation (CV, CV = S. D. /mean) of the rDNA copy number in a single strain was calculated to be less than 6%.

## 4 DISCUSSION

Some rDNA copy number estimation methods were previously developed. These approaches include pulsed‐field gel electrophoresis followed by rDNA‐specific Southern blotting.^15^, ^17^ rDNA‐targeted qPCR^19^, ^24^ or droplet digital PCR,^25^ and whole genome sequencing.^16^, ^18^ Recent studies have used qPCR to estimate rDNA copy numbers because qPCR technology offers the fast and reliable quantification of any target sequence in a sample. It also has many advantages over alternative methods, such as low consumable and instrumentation costs, fast assay development times and high sensitivity.^26^ In the present study, we developed a measurement method using qPCR that estimates the rDNA copy number of *T. interdigitale* by designing ITS and MDR primer sequences.

The genomes of most organisms contain multiple copies of rDNA, and the rDNA copy number varies among strains. Previous studies showed that rDNA copy numbers varied between 1 and 15 in Bacteria and Archaea,^27^, ^28^, ^29^ between 39 and 19,300 in animals, and between 150 and 26 048 in plants.^30^ rDNA repeats in the fungal genome were reported to be between 14 and 1442 using 91 species.^16^ Investigations on single fungal species revealed that rDNA copy numbers varied among strains. The rDNA copy numbers of *L. maculans*,^17^ *S. cerevisiae* ^18^ and *A. fumigatus* ^19^ varied between fungal strains. This is a first report of the rDNA copy number in the dermatophytes, focusing on *T. interdigitale*. The 64 strains showed a range of between 24 and 116 copies in a genome of *T. interdigitale*. This range of rDNA was within that of other fungal rDNA copy numbers, and rDNA copy number variations were also observed among the strains of *T. interdigitale*.

As a result of the homogeneity evaluation of rDNA copy numbers using the single‐spore culture of *T. interdigitale* strains, the CV of the rDNA copy number from each strain was less than 6. The standard curve of ITS was created using triplicate measurements at each point. The CV of each point in the standard curve was calculated as between 3 and 7 using the formula of the regression line and Ct value at each point. Thus, CV from the rDNA copy number in a single strain was within those from qPCR measurements. No significant change was detected by this qPCR measurement in the case of short‐term cultures, which was consistent with previous findings obtained using *A. fumigatus*. The rDNA copy number of *A. fumigatus* remained stable under various environmental conditions, such as morphology, growth temperature, culture age, antifungal exposure and animal infection model organ site (lungs vs kidneys).^19^

No correlations were observed between the rDNA copy number and colony colour, colony morphology or the NTS type in the 64 clinical isolates of *T. interdigitale*. We previously classified these 64 isolates into 15 NTS types.^20^ The NTS regions of rDNA accumulate high degrees of sequence variations, and the detection of these variations is the most frequently used method for the strain typing of dermatophytes.^5^ Present study showed rDNA copy numbers varied more than the NTS type. These results suggest the potential of rDNA copy numbers as a sensitive molecular marker for detecting variations among strains of *T. interdigitale* and may help distinguish between recurrences due to the original strain and a new strain.

The direct KOH examination and fungal culture are gold standards for the diagnosis and evaluations of the outcomes of dermatophytosis. However, it is generally impossible to evaluate the viability of fungi in specimens. Several approaches have been developed to overcome this issue using molecular biological techniques.^31^, ^32^ Among them, the evaluation method for viability was developed using qPCR targeting the ITS region of rDNA, which revealed a correlation between ITS DNA and CFU 48 hours after death in *T. interdigitale*.^21^ In addition, the amounts of rDNA in toe nail and skin specimens from patients with tinea pedis and tinea unguium were quantified using the ITS region of rDNA targeted by qPCR.^33^, ^34^, ^35^ By combining the investigation of rDNA copy numbers with the quantification of rDNA amounts in specimens, it will be possible to estimate the amount of *T. interdigitale* in affected sites. This finding may provide a more detailed understanding of the mechanisms underlying the attenuation and recalcitrance of dermatophytosis caused by *T. interdigitale*. Therefore, further studies that focus on the rDNA copy numbers of frequently isolated pathogenic dermatophyte species other than *T. interdigitale*, such as *T. rubrum*, *T. tonsurans*, and *Microsporum canis*, are needed.

In conclusion, we herein developed a method to estimate rDNA copy numbers in the genome of *T. interdigitale* using qPCR. This is the first study to investigate the copy number of the rDNA subunit in a genome of *T. interdigitale*. The rDNA copy number of *T. interdigitale* varied among strains, from 24 to 116 copies per genome, and the rDNA copy number of each strain appeared to be homogeneous.

## CONFLICT OF INTEREST

Tomoyuki Iwanaga is an employee of POLA Chemical Industries, Inc (Japan).

## AUTHOR CONTRIBUTION

**Tomoyuki Iwanaga:** Conceptualization (lead); Data curation (lead); Formal analysis (lead); Investigation (lead); Methodology (lead); Writing‐original draft (lead); Writing‐review & editing (lead). **Kazushi Anzawa:** Data curation (supporting); Formal analysis (supporting); Investigation (supporting); Resources (supporting). **Takashi Mochizuki:** Resources (lead); Supervision (lead); Writing‐original draft (supporting); Writing‐review & editing (supporting).

## References

[1] Priestley H . Ringworm and allied parasitic skin diseases in Australia. Med J Aust. 1917;2:471‐475.

[2] Havlickova B , Czaika VA , Friedrich M . Epidemiological trends in skin mycoses worldwide. Mycoses. 2008;51:2‐15. 18783559 10.1111/j.1439-0507.2008.01606.x

[3] Zhan P , Liu W . The changing face of dermatophytic infections worldwide. Mycopathologia. 2017;182:77‐86. 27783316 10.1007/s11046-016-0082-8

[4] Kanbe T . Molecular approaches in the diagnosis of dermatophytosis. Mycopathologia. 2008;166:307‐317. 18481195 10.1007/s11046-008-9107-2

[5] Mochizuki T , Takeda K , Anzawa K . Molecular markers useful for intraspecies subtyping and strain differentiation of dermatophytes. Mycopathologia. 2017;182:57‐65. 27456819 10.1007/s11046-016-0041-4

[6] Jackson CJ , Barton RC , Evans EG . Species identification and strain differentiation of dermatophyte fungi by analysis of ribosomal‐DNA intergenic spacer regions. J Clin Microbiol. 1999;37:931‐936. 10074504 10.1128/jcm.37.4.931-936.1999 PMC88627

[7] Mochizuki T , Tanabe H , Kawasaki M , Ishizaki H , Jackson CJ . Rapid identification of *Trichophyton tonsurans* by PCR‐RFLP analysis of ribosomal DNA regions. J Dermatol Sci. 2003;32:25‐32. 12788526 10.1016/s0923-1811(03)00030-6

[8] Shin J‐H , Sung J‐H , Park S‐J , et al. Species identification and strain differentiation of dermatophyte fungi using polymerase chain reaction amplification and restriction enzyme analysis. J Am Acad Dermatol. 2003;48:857‐865. 12789175 10.1067/mjd.2003.491

[9] White TJBT , Lee S , Taylor J . Amplification and direct sequencing of fungal ribosomal RNA genes for phylogenetics In: Innis MA, Gelfand DH, Sninsky JJ, White TJ, eds. PCR Protocols: A Guide to Methods and Applications. San Diego, CA: Academic Press; 1990:315‐322.

[10] Makimura K , Mochizuki T , Hasegawa A , Uchida K , Saito H , Yamaguchi H . Phylogenetic classification of *Trichophyton mentagrophyte*s complex strains based on DNA sequences of nuclear ribosomal internal transcribed spacer 1 regions. J Clin Microbiol. 1998;36:2629‐2633. 9705405 10.1128/jcm.36.9.2629-2633.1998 PMC105175

[11] Mochizuki T , Kawasaki M , Ishizaki H , Makimura K . Identification of several clinical isolates of dermatophytes based on the nucleotide sequence of internal transcribed spacer 1 (ITS 1) in nuclear ribosomal DNA. J Dermatol. 1999;26:276‐281. 10380427 10.1111/j.1346-8138.1999.tb03472.x

[12] Makimura K , Tamura Y , Mochizuki T , et al. Phylogenetic classification and species identification of dermatophyte strains based on DNA sequences of nuclear ribosomal internal transcribed spacer 1 regions. J Clin Microbiol. 1999;37:920‐924. 10074502 10.1128/jcm.37.4.920-924.1999 PMC88625

[13] Gräser Y , El Fari M , Vilgalys R , et al. Phylogeny and taxonomy of the family Arthrodermataceae (dermatophytes) using sequence analysis of the ribosomal ITS region. Med Mycol. 1999;37:105‐114. 10361266

[14] Graser Y , Kuijpers AF , Presber W , De Hoog GS . Molecular taxonomy of *Trichophyton mentagrophytes* and *T tonsurans* . Med Mycol. 1999;37:315‐330. 10520156 10.1046/j.1365-280x.1999.00234.x

[15] Maleszka R , Clark‐Walker GD . Yeasts have a four‐fold variation in ribosomal DNA copy number. Yeast. 1993;9:53‐58. 8442387 10.1002/yea.320090107

[16] Lofgren LA , Uehling JK , Branco S , Bruns TD , Martin F , Kennedy PG . Genome‐based estimates of fungal rDNA copy number variation across phylogenetic scales and ecological lifestyles. Mol Ecol. 2019;28:721‐730. 30582650 10.1111/mec.14995

[17] Howlett BJ , Rolls BD , Cozijnsen AJ . Organisation of ribosomal DNA in the ascomycete *Leptosphaeria maculans* . Microbiol Res. 1997;152:261‐267. 9352662 10.1016/S0944-5013(97)80038-9

[18] Liti G , Carter DM , Moses AM , et al. Population genomics of domestic and wild yeasts. Nature. 2009;458:337‐341. 19212322 10.1038/nature07743 PMC2659681

[19] Herrera ML , Vallor AC , Gelfond JA , Patterson TF , Wickes BL . Strain‐dependent variation in 18S ribosomal DNA copy numbers in *Aspergillus fumigatus* . J Clin Microbiol. 2009;47:1325‐1332. 19261786 10.1128/JCM.02073-08 PMC2681831

[20] Wakasa A , Anzawa K , Kawasaki M , Mochizuki T . Molecular typing of *Trichophyton mentagrophytes* var. *interdigitale* isolated in a university hospital in Japan based on the non‐transcribed spacer region of the ribosomal RNA gene. J Dermatol. 2010;37:431‐440. 20536648 10.1111/j.1346-8138.2010.00809.x

[21] Iwanaga T , Anzawa K , Mochizuki T . Quantification of dermatophyte viability for evaluation of antifungal effect by quantitative PCR. Mycopathologia. 2014;177:241‐249. 24760383 10.1007/s11046-014-9745-5

[22] Fachin AL , Ferreira‐Nozawa MS , Maccheroni W , Martinez‐Rossi NM . Role of the ABC transporter TruMDR2 in terbinafine, 4‐nitroquinoline N‐oxide and ethidium bromide susceptibility in *Trichophyton rubrum* . J Med Microbiol. 2006;55:1093‐1099. 16849730 10.1099/jmm.0.46522-0

[23] Makimura K , Murayama SY , Yamaguchi H . Detection of a wide range of medically important fungi by the polymerase chain reaction. J Med Microbiol. 1994;40:358‐364. 8176723 10.1099/00222615-40-5-358

[24] Black J , Dean T , Byfield G , Foarde K , Menetrez M . Determining fungi rRNA copy number by PCR. J Biomol Tech. 2013;24:32‐38. 23543828 10.7171/jbt.13-2401-004 PMC3523570

[25] Salim D , Bradford WD , Freeland A , et al. DNA replication stress restricts ribosomal DNA copy number. PLoS Genet. 2017;13:e1007006. 28915237 10.1371/journal.pgen.1007006 PMC5617229

[26] Rubio‐Piña J , Quiroz‐Moreno A , Sánchez‐Teyer LF . A quantitative PCR approach for determining the ribosomal DNA copy number in the genome of *Agave tequila* Weber. Electron J Biotechnol. 2016;22:9‐15.

[27] Loughney K , Lund E , Dahlberg JE . tRNA genes are found between 16S and 23S rRNA genes in *Bacillus subtilis* . Nucleic Acids Res. 1982;10:1607‐1624. 6280153 10.1093/nar/10.5.1607 PMC320553

[28] Rainey FA , Ward‐Rainey NL , Janssen PH , Hippe H , Stackebrandt E . Clostridium paradoxum DSM 7308T contains multiple 16S rRNA genes with heterogeneous intervening sequences. Microbiology. 1996;142(Pt 8):2087‐2095. 8760921 10.1099/13500872-142-8-2087

[29] Liao D . Gene conversion drives within genic sequences: concerted evolution of ribosomal RNA genes in bacteria and archaea. J Mol Evol. 2000;51:305‐317. 11040282 10.1007/s002390010093

[30] Prokopowich CD , Gregory TR , Crease TJ . The correlation between rDNA copy number and genome size in eukaryotes. Genome. 2003;46:48‐50. 12669795 10.1139/g02-103

[31] Mirhendi H , Motamedi M , Makimura K , Satoh K . Development a diagnostic pan‐dermatophyte TaqMan probe real‐time PCR assay based on beta tubulin gene. Mycoses. 2016;59:520‐527. 27071371 10.1111/myc.12502

[32] Verrier J , Monod M . Diagnosis of dermatophytosis using molecular biology. Mycopathologia. 2017;182:193‐202. 27480761 10.1007/s11046-016-0038-z

[33] Miyajima Y , Satoh K , Uchida T , et al. Rapid real‐time diagnostic PCR for *Trichophyton rubrum* and *Trichophyton mentagrophytes* in patients with tinea unguium and tinea pedis using specific fluorescent probes. J Dermatol Sci. 2013;69:229‐235. 23287391 10.1016/j.jdermsci.2012.11.589

[34] Iwanaga T , Ushigami T , Anzawa K , Mochizuki T . Pathogenic dermatophytes survive in nail lesions during oral terbinafine treatment for tinea unguium. Mycopathologia. 2017;182:673‐679. 28281037 10.1007/s11046-017-0118-8 PMC5500682

[35] Iwanaga T , Ushigami T , Anzawa K , Mochizuki T . Viability of pathogenic dermatophytes during a 4‐week treatment with 1% topical luliconazole for tinea pedis. Med Mycol. 2020;58:401‐403. 31111903 10.1093/mmy/myz056 PMC7108760

